# Commentary: Coordinated infraslow neural and cardiac oscillations mark fragility and offline periods in mammalian sleep

**DOI:** 10.3389/fphys.2017.00847

**Published:** 2017-11-10

**Authors:** Mauro Manconi, Alessandro Silvani, Raffaele Ferri

**Affiliations:** ^1^Sleep and Epilepsy Center, Neurocenter of Southern Switzerland, Civic Hospital (EOC) of Lugano, Lugano, Switzerland; ^2^Department of Neurology, Bern University Hospital, Bern, Switzerland; ^3^Department of Biomedical and Neuromotor Sciences, University of Bologna, Bologna, Italy; ^4^Sleep Research Centre, Department of Neurology I.C., Oasi Institute for Research on Mental Retardation and Brain Aging (IRCCS), Troina, Italy

**Keywords:** sleep, EEG, heart rate, humans, mice

We read with interest the paper by Lecci et al. (2017), who showed oscillations of the electroencephalographic (EEG) spectral power in the sigma band (10–15 Hz) during non-rapid-eye-movement (NREM) sleep at frequencies in the infra-slow range (ISO = 0.001–0.1 Hz). The occurrence of this rhythm (sigma-ISO) in human subjects and mice, and its correlation with autonomic and behavioral components suggest that it reflects a fundamental physiological mechanism.

One of the keys to this fundamental mechanism may be that, based on its frequency of occurrence and its segregation in NREM sleep, the sigma-ISO bears a similarity to the cyclic alternating pattern (CAP). CAP is a visually-detectable oscillation in brain activity occurring during NREM sleep with periodicity of 20–40 s (Terzano and Parrino, 1991). CAP sequences consist of an activation component (phase A) followed by a deactivation component (phase B).

One important novelty of the study by Lecci et al. (2017) concerns the discovery of sigma-ISO in rodents. Since CAP has never been demonstrated in rodents, the sigma-ISO might represent a basic correlate of CAP translatable among species. Another important novelty was the demonstration of ISO during the whole NREM sleep of human subjects, including the so-called “non-CAP period” when sleep seems to be stable, without any visually detectable ISO of the EEG background.

In this light, we thought that it could be useful to review and summarize the similarities and dissimilarities between the sigma-ISO and CAP (Table 1).

**Table 1:** Similarities and differences between oscillations of the EEG power in the sigma frequency band (sigma-ISO, infra-slow oscillation) and the Cyclic Alternating Pattern (CAP).

	**Sigma-ISO**	**CAP**	
		**Phase A**	**Phase B**
Frequency	0.02 Hz	0.04 Hz	
Sleep Stage	Whole NREM	NREM (CAP-period)	
N2	↑↑	↑↑	
N3	↑	↑	
REM	NO	NO	
Mapping	Parietal	Fronto-mesial and/or parieto-occipital	?
Spindles Density	High correlation	Low	High
Sensory Threshold	High	Low	High
Hippocampal Ripples	High	?	?
Cognitive Process	High Correlation	High Correlation	
Heart Rate	↑	↑	↓

## Frequency of oscillation

The frequency of the sigma-ISO in young humans (mean age 22.5 years) is 0.02 Hz, which corresponds to a period of around 50 s. The typical CAP period in healthy young humans is approximately 25 s (0.04 Hz; Ferri et al., 2006), half the period of the sigma-ISO. The sigma-ISO might thus be akin to a subharmonic of CAP, with a possible phase-locked synchronization such that one sigma-ISO cycle contains two CAP cycles.

## Sleep stages

Lecci et al. (2017) claimed that one of the reasons why CAP is different from sigma-ISO is that CAP “*occurs throughout all sleep stages with wide variations in its spectral composition*.” Indeed, CAP disappears in rapid-eye-movement (REM) sleep, and is almost absent in NREM sleep immediately following a REM period, which was in fact named “non-CAP period.” Moreover, the wide variation in the spectral EEG composition of CAP regards only its phase A, which, for this reason, was subdivided into three different subtypes (A1, A2, and A3), depending on the balance between slow and rapid EEG components, but does not involve CAP phase B, which is highly homogeneous with low-voltage rapid EEG waves.

## Sleep spindles

The power of the sigma-ISO in humans correlates with the density of sleep spindles, in particular of the “fast” spindles with higher frequency (13–15 Hz), which are generated by the thalamocortical network. Spindles tend to occur during the CAP phase B or during non-CAP periods (Ferri et al., 2005a). This raises the hypothesis that sigma-ISO is synchronized with CAP phase B. Empirical tests of this hypothesis are possible, and would clarify the relationship between these two fundamental ISO rhythms.

## Regional specificity

The sigma-ISO has maximum power over parietal EEG derivations in humans. To our knowledge, a detailed mapping of the CAP oscillation has been computed only for the CAP phase A, with a peak in the fronto-mesial cortex for the EEG delta band and over the parieto-occipital cortex for the EEG alpha band, whereas a structured mapping of CAP phase B has never been carried out. However, several studies, also by using high-density EEG analysis, identified the temporo-parietal areas as the main source of the fast component of spindles (Ferri et al., 2005b).

## Susceptibility to external stimuli

The arousal probability of mice is higher during periods of declining sigma-ISO power and lower when sigma-ISO power rises. It has been shown that in humans, resilience to external stimuli is higher in CAP phase B and lower in CAP phase A (Terzano and Parrino, 1991). Again, this would be consistent with the hypothesis of a synchronization between sigma-ISO and CAP phase B.

## Hippocampal ripples and memory consolidation

Hippocampal ripples are high-frequency field oscillations occurring during slow-wave sleep and non-exploratory wake states in humans and laboratory animals. During sleep, ripples trigger synaptic modifications favoring neuronal hippocampal and neocortical plasticity, playing a crucial role in the information transfer between hippocampus and cortex and participating in memory consolidation (Girardeau and Zugaro, 2011).

The sigma-ISO is coordinated with hippocampal ripples in mice. No studies have addressed the relationship between CAP and hippocampal ripples in humans. However, several studies highlighted the importance of CAP for cognitive function, including memory consolidation (Aricò et al., 2010; Esposito and Carotenuto, 2010).

## Heart rate changes

Lecci et al. (2017) showed that the heart rate increases/declines together with the sigma-ISO with a lag of about 5 s in humans. Peaks in heart rate are also coupled with CAP phase A (Ferri et al., 2000; Kondo et al., 2014). This might suggest a synchronization between the sigma-ISO and CAP phase A, in contrast with the previous arguments based on sleep spindles and susceptibility to external stimuli.

The scoring of CAP yields objective indexes of sleep quality. Understanding whether the sigma-ISO is indeed synchronized with CAP, and, if so, whether synchronization occurs with CAP phase A or B, would pave the way to develop better sleep quality indexes based jointly on sigma-ISO and CAP, and to predict the effects exerted by the modulation of CAP on sigma-ISO and vice versa.

Possible steps in the research agenda might be: to replicate the results of Lecci et al. (2017); to ascertain whether sigma-ISO and CAP are synchronized; to verify whether specific pathologies associated with a quantitative alteration of CAP also affect sigma-ISO.

A coincidence or a significant synchronization between sigma-ISO and CAP would open the way to a novel area of research in integrative physiology, where sigma-ISO and CAP might integrate neural, cardiovascular, and somatic motor control during sleep at frequencies bridging the fast EEG phenomena and the slow circadian oscillations.

## Author contributions

Conception of the work: MM, AS, and RF. Interpretation of data for the work: MM, AS, and RF. Drafting the work: MM. Revising the work critically for important intellectual content: AS and RF. Final approval of the version to be published: MM, AS, and RF. Agreement to be accountable for all aspects of the work in ensuring that questions related to the accuracy or integrity of any part of the work are appropriately investigated and resolved: MM, AS, and RF.

### Conflict of interest statement

The authors declare that the research was conducted in the absence of any commercial or financial relationships that could be construed as a potential conflict of interest.
